# Characteristic DNA methylation profiles of chorionic villi in recurrent miscarriage

**DOI:** 10.1038/s41598-022-15656-y

**Published:** 2022-07-27

**Authors:** Yosuke Matsumoto, Keiko Shinjo, Shoko Mase, Masaki Fukuyo, Kosuke Aoki, Fumiko Ozawa, Hiroyuki Yoshihara, Shinobu Goto, Tamao Kitaori, Yasuhiko Ozaki, Satoru Takahashi, Atsushi Kaneda, Mayumi Sugiura-Ogasawara, Yutaka Kondo

**Affiliations:** 1 Department of Obstetrics and Gynecology, Nagoya City University Graduate School of Medical Sciences, Nagoya, 467-8601 Japan; 2 Division of Cancer Biology, Nagoya University Graduate School of Medicine, Nagoya, 466-8550 Japan; 3 Department of Molecular Oncology, Graduate School of Medicine, Chiba University, Chiba, 260-8670 Japan; 4 Department of Neurosurgery, Nagoya University Graduate School of Medicine, Nagoya, 466-8550 Japan; 5 Department of Experimental Pathology and Tumor Biology, Nagoya City University Graduate School of Medical Sciences, Nagoya, 467-8601 Japan

**Keywords:** Health care, Intrauterine growth, Developmental biology, Embryology

## Abstract

Dysregulation of transcriptional programs that are tightly regulated by DNA methylation during placental and fetal development at different gestational stages, may cause recurrent miscarriage. Here, we examined genome-wide DNA methylation in chorionic villi and decidual tissues from patients suffering RM and from healthy women who had undergone artificial abortion (n = 5 each). We found that 13,426 and 5816 CpG sites were differentially methylated in chorionic villi and decidua, respectively. DNA methylation profiles of chorionic villi, but not decidua, in RM patients was clearly distinct from AA controls. Among the differentially methylated genes, the enhancer region of *SPATS2L* was significantly more highly methylated in RM patients (n = 19) than AA controls (n = 19; mean methylation level, 52.0%-vs.-28.9%, *P* < 0.001), resulting in reduced expression of SPATS2L protein in the former. Functionally, depletion of *SPATS2L* in extravillous trophoblast cells decreased their invasion and migration abilities. Our data indicate that particularly the chorionic villi in RM patients exhibit distinct DNA methylation profiles compared with normal pregnancies and that this changed DNA methylation status may impede the progression of embryo development via the altered expression of genes such as *SPATS2L* in the villi.

## Introduction

Recurrent miscarriage (RM), the frequency of which is approximately 0.7% of all pregnancies, is defined as three or more consecutive miscarriages before week 22 of gestation, according to the guidelines of the European Society for Human Reproduction and Embryology^1,2^. Because RM is one of the most distressing complications of pregnancy, and the frequency increases with maternal age^3^, understanding the mechanisms responsible for this has been very actively investigated. In addition to antiphospholipid syndrome (APS), uterine anomalies and parental chromosomal abnormalities, embryonic aneuploidy was found to be the most common cause of RM, accounting for 41% of cases^4^. However, causes of RM remain unknown in a quarter of cases; the frequency of unexplained RM with a normal embryonic karyotype was 5–25% according to a recent microarray analysis^5^. Because the prognosis of RM patients who had previously had a normal embryonic karyotype is poorer than that of patients with previous embryonic aneuploidy, elucidating the causes of RM is a matter of immediate concern^6^.

DNA methylation in gene regulatory regions, such as enhancers and promoters, is closely associated with gene expression, including of lineage-specific genes^7,8^. During early embryogenesis after fertilization, a dynamic epigenetic reprogramming ensures the correct development of the embryos^9,10^. Recent studies documented altered DNA methylation in certain sets of genes, such as imprinting loci, in the chorionic villi of RM, or sporadic miscarriage after in vitro fertilization^11–13^. In addition, a genome-wide DNA methylation analysis in decidua revealed that hypomethylation of CAMP Responsive Element Binding Protein 5 (*CREB5*) appeared to be a risk factor for recurrent pregnancy loss, by causing dysfunction of trophoblast cells^14^. These studies indicate that aberrant epigenetic regulation of maternal (decidua) and fetal (villi) tissue is involved in early embryogenesis. However, information on aberrant DNA methylation in association with RM is still limited. Many questions remain, for example, (1) Are there any specific aberrations in DNA methylation profiles in decidua and chorionic villi in RM patients? (2) Are there any specific common genes that show aberrant DNA methylation in either of these tissues in RM patients? (3) How does DNA methylation status in such genes change during early pregnancy? Because chorionic villi and maternal decidua have different DNA methylation profiles^15^, more precise analysis of DNA methylation status in RM is required for better understanding of aberrant epigenetic regulation during pregnancy.

In the current study, we investigated genome-wide DNA methylation profiles in both chorionic villi and decidual tissues from the products of conception (POC) in patients with euploid RM were detected, compared with the same tissues from women with artificial abortions (AAs) as controls of the same gestational week. Our data establish characteristic genome-wide aberrant DNA methylation programs involved in RM processes.

## Materials and methods

### Patients

Patients with a history of unexplained RM who visited Nagoya City University Hospital between 2012 and 2017 were enrolled. Before subsequent pregnancy, all participants underwent systematic examination, including 4D-ultrasound sonography and/or hysterosalpingography, chromosome analysis of both partners, blood tests for hypothyroidism and diabetes mellitus. They were screened for antiphospholipid antibodies, lupus anticoagulant (LA) detected by diluted activated partial prothrombin time and LA detected by diluted Russell’s viper venom time, as well as β2 glycoprotein I-dependent anticardiolipin antibody. Patients with APS, abnormal chromosomes in either partner, or with uterine anomalies were excluded from the study. Finally, 19 patients were enrolled in the present investigation.

Subsequent pregnancies of the patients were followed up. Gestational weeks were calculated from basal body temperature charts. Ultrasonography was performed once a week from 4 to 8 weeks of gestation. Gestational age was determined by measuring the size of the fetus. Dilation and curettage were performed on the patients diagnosed as having a miscarriage. Part of the villi was cultured, and the cells were harvested after 6–22 days later for chromosomal analysis. Aborted POC were karyotyped using a standard G-banding technique and patients with abnormal chromosome karyotypes were excluded.

Chorionic villi and decidua were carefully separated under an anatomical microscope and samples of each were frozen at − 80 °C. Part of each sample was fixed with 10% buffered formalin and paraffin embedded for immunohistochemistry.

Control samples were obtained from 19 pregnant women with no history of miscarriage who had undergone an artificial abortion procedure in the first trimester at Kato Lady’s Clinic, Asamoto Lady’s Clinic, or Mizuno Lady’s Clinic, Nagoya, Japan. We selected AA controls without a past history of hypothyroidism and diabetes mellitus for further analysis. Gestational age was determined by measuring of the size of the fetus by ultrasonography. For this study, we analyzed age- and gestational weeks-matched AA controls.

Five patients with RM and five matched controls who underwent AA were selected for genome-wide DNA methylation analysis. All chorionic villi were confirmed as karyotypically normal by array comparative genomic hybridization (CGH) analysis using SurePrint G3 Human CGH microarrays (Agilent Technologies, Santa Clara, CA, USA).

Collection of the samples for this study was approved by the Research Ethics Committee of Nagoya City University Medical School and collaborating clinics. Written informed consent was obtained from all patients and controls. All research was performed in accordance with relevant guidelines and regulations.

### DNA extraction from samples

DNA from chorionic villi and decidua was extracted using DNeasy Blood and Tissue Kits (Qiagen, Hilden, Germany) according to the manufacturer’s protocol, and all extracted DNA samples were kept at − 20 °C.

### Genome-wide DNA methylation analysis

Genomic DNA (250 ng) from villi and decidua of five patients with RM and five controls after AA was treated with bisulfite using an EZ DNA Methylation Kit (Zymo Research, Irvine, CA, USA). Genome-wide DNA methylation analysis was performed using the Infinium Human Methylation450 BeadChip, according to the manufacture’s protocol (Illumina, San Diego, CA, USA). The raw image intensities of the hybridized arrays were quantified by an iScan SQ scanner (Illumina), and the raw image intensity data were processed by GenomeStudio software (Illumina). The percentage of methylated cytosines at CpG loci of each probe was calculated as a β value, varying from 0 (completely unmethylated) to 1 (completely methylated). For quality control, probes with missing values, genes on the X and Y chromosomes, and with the SNPs at minor allele frequency > 0.1 were eliminated.

Probes with a difference between RM patients and AA controls in the average β-value (|Δβ|) > 0.1 were defined as differentially methylated probes (DMPs). The CpG score of the 500 bp region around each probe (± 250 bp) was calculated based on previous reports^16,17^. Probes on promoter regions located around the transcriptional start sites (TSS ± 1500 bp) and probes on enhancer regions were extracted according to the Infinium HumanMethylation450 BeadChip annotation file.

Unsupervised two-way hierarchical cluster analysis of the DMPs of the chorionic villi and decidua was performed according to the region of the probes (all regions, promoter regions, enhancer regions, and gene bodies), using the Genespring GX 14.9 software (Agilent Technologies). This cluster analysis was performed with Ward’s method and Euclidean distance. When multiple probes were designed for one gene, a single representative probe with has the highest CpG score was selected. The optimal number of clusters was analyzed by consensus clustering using the ConsensusClusterPlus package in R.

### Gene ontology analysis

Gene annotation enrichment analysis of the differentially methylated genes on the promoter and enhancer regions was performed using the Functional Annotation tool available at DAVID 6.8 Bioinformatics Resources (https://david.ncifcrf.gov/home.jsp).

### Targeted DNA methylation analysis

DNA from chorionic villi of a total of 19 patients with RM and 19 AA controls, including the 5 samples used for genome-wide methylation analysis and the remaining 14 samples, was used for targeted DNA methylation analysis. DNA was treated with bisulfite using an EpiTect Plus Bisulfite Kit (Qiagen). PCR primers for pyrosequencing were designed by Pyromark Assay Design 2.0 (Qiagen) (Supplementary Table S1). By using methylation control samples (0%, 50%, 100%) from the Epitect control DNA set (Qiagen), the quality of each pyrosequencing assay was confirmed. The methylation level was analyzed by pyrosequencing technology (PyroMark Q24, Qiagen) and the mean DNA methylation level of CpGs was calculated for each sample.

### Immunohistochemical staining analysis

Samples of chorionic villi samples from RM patients (n = 12) and AA controls (n = 14), including the samples that underwent genome-wide DNA methylation analysis, were immunostained with anti-SPATS2L antibody (16,938-AP, Proteintech, Rosemont, USA) diluted 1:200. To this end, the samples were dehydrated and embedded in paraffin, cut into 3 mm sections and placed on MAS-coated glass slides. Immunostaining was performed with the Leica Bond-Max automation and Leica Refine detection kits (Leica Biosystems, Bannockburn, IL, USA) according to the manufacturer’s protocol. The protocol included in situ deparaffinization and high-pH epitope retrieval for 25 min, incubation with primary antibody for 30 min, polymer for 8 min, and DAB as the chromogen for 10 min, followed by a 5-min hematoxylin counterstaining. The negative control for immunohistochemical analysis was incubating with normal rabbit IgG (PM035, MBL, Tokyo, Japan) and with secondary antibody or without any first antibody. The number of cytotrophoblast cells stained with SPATS2L per 5 high-power-fields (HPF) was counted.

### Cell culture

The first trimester trophoblast cell line, HTR8/SVneo, was purchased from the American Type Culture Collection (ATCC). Cells were cultured in RPMI-1640 (Gibco, MA, USA) containing 10% fetal bovine serum (Gibco) and 1% penicillin–streptomycin (Gibco) at 37 °C in a humidified incubator with 5% CO_2_.

### RNA interference

Cells were treated with two independent siRNAs targeting *SPATS2L* (50 nmol/L, #1 and #2, Supplementary Table S1) or negative control siRNA (50 nmol/L, Silencer Select Negative Control #1 siRNA, 4,390,844, Thermo Fisher Scientific, Waltham, USA) using Lipofectamine 3000 (Thermo Fisher Scientific) according to the manufacturer's protocol. RNA was extracted 48 h and protein 72 h after siRNA treatment.

### Quantitative RT-PCR analysis

Total RNA from the HTR-8/SVneo cell line was extracted after siRNA treatment using RNeasy Mini Kits (Qiagen), followed by reverse-transcription using Prime Script RT Master Mix (Takara, Kusatsu, Japan). SYBR Green qPCR (TOYOBO, Osaka, Japan) was performed at least in duplicate for the target genes. Expression levels of the *SPATS2L* gene were normalized to *GAPDH*. The primer sequences used for RT-PCR assays are shown in Supplementary Table S1.

### Western blotting

Cell lysates were prepared from cells after siRNA treatment. A total of 20 μg of protein was separated by 10% SDS/PAGE gels, transferred to nitrocellulose membranes and incubated with the following primary antibodies: rabbit polyclonal anti-SPATS2L (16,938-AP, Proteintech, Rosemont, USA) and mouse monoclonal anti-GAPDH (#97,196, Cell Signaling Technology, Danvers, MA, USA). Secondary antibodies were HRP-linked anti-rabbit IgG (#7074, Cell Signaling Technology) and HRP-linked anti-mouse IgG (#7076, Cell Signaling Technology). The density of bands was quantified by ImageJ software (https://imagej.nih.gov/ij/).

### Transwell invasion and cell migration assays

Matrigel invasion assays were performed using Corning BioCoat Matrigel Invasion Chambers (8-μm pore, Thermo Fisher Scientific) and Corning BioCoat Control Inserts (without Matrigel, 8-μm pore, Thermo Fisher Scientific). The lower chambers were filled with RPMI-1640 containing 10% FBS. After 48 h of siRNA treatment, 1 × 10^5^ HTR-8/SVneo cells were seeded into the upper chamber without FBS. After a further 24 h, cells attached to the bottom membrane (invasive cells) were stained with Diff-Quick stain (Sysmex, Kobe, Japan) and counted in at least 3 different fields of view. The invasion rate was calculated as (the number of cells on the bottom membrane in the Matrigel chambers) divided by (the number of cells on the bottom membrane in the control chambers).

For cell migration analysis, HTR-8/SVneo cells were seeded into 24-well plates and cultured to confluence after 48 h of siRNA treatment. A straight scratch was made in the cell monolayer using a p1000 pipette tip. Cells were gently washed with PBS twice and cultured for a further 24 h. Images were captures at 0 h (immediately after the scratch was made) and after 24 h using the IncuCyte Live Cell Analysis System (Sartorius, Goettingen, Germany). The area without cells was measured using ImageJ software. The migration rate was calculated as (the area without cells at 0 h minus that at 24 h) divided by (the area without cells at 0 h).

### Statistical analysis

Statistical analyses were performed using SPSS software version 23.0 (IBM, Chicago, IL, USA). The statistical significance of differences between 2 groups was analyzed by the Mann–Whitney’s U test. Spearman’s correlation coefficient was used to assess whether there was a correlation between gestational age and DNA methylation level. All reported *P* values were two-sided and *P* < 0.05 was considered statistically significant.

## Results

### Genome-wide DNA methylation analysis in chorionic villus and decidual tissues from RM patients and AA controls

First, genome-wide DNA methylation profiles were established using POC tissues from RM patients (n = 5) and AA controls (n = 5) whose karyotypes were diagnosed as normal according to the previously reported criteria^18^. RM patients had suffered a mean of 4.8 miscarriages (range 3–7 times) (Table 1, Supplementary Table S2). All the RM patients gave birth at least once after several miscarriages (Supplementary Fig. S1). At the time of sampling, there were no significant differences in maternal ages and mean gestational weeks between RM and AA (34.8 ± 3.1 vs. 32.6 ± 7.7 years, *P* = 0.62 and 7.2 ± 0.8 vs. 7.1 ± 0.8 weeks, *P* = 0.80, respectively). Using a stereoscopic microscope, samples were separated into chorionic villus and decidual tissues just after the miscarriage or abortion, and were subsequently examined for DNA methylation profiles using Illumina Infinium HumanMethylation450 BeadChip technology.

**Table 1:** Clinical background of RM patients and AA controls for genome-wide DNA methylation analysis and pyrosequencing.

	Genome-wide analysis			Pyrosequencing analysis*		
	RM Patients (n = 5)	AA Controls (n = 5)	*P* value	RM Patients (n = 19)	AA Controls (n = 19)	*P* value
Age	34.8 ± 3.1	32.6 ± 7.7	0.62	34.5 ± 3.6	32.4 ± 6.2	0.23
Gestational weeks	7.2 ± 0.8	7.1 ± 0.8	0.80	7.0 ± 1.0	7.8 ± 1.3	0.053
Miscarriages	4.8 (3–7)	0 (0)	< 0.01	3.7 (3–7)	0.32 (0–3)	< 0.01
Previous live births	0.2 (0–1)	2.0 (0–5)	0.13	0.42 (0–1)	1.6 (0–5)	< 0.01
Male/female ratio of POC	4:1	1:4	< 0.01	10:9	8:9 not tested:2	0.48

Age and gestational weeks are given as mean ± standard deviation (SD).

Miscarriages and previous live births are given as mean number (range).

*RM* recurrent miscarriage; *AA* artificial abortion; *POC* products of conception.

*Samples include five RM and five AA used for the genome-wide analysis.

**Figure 1:**
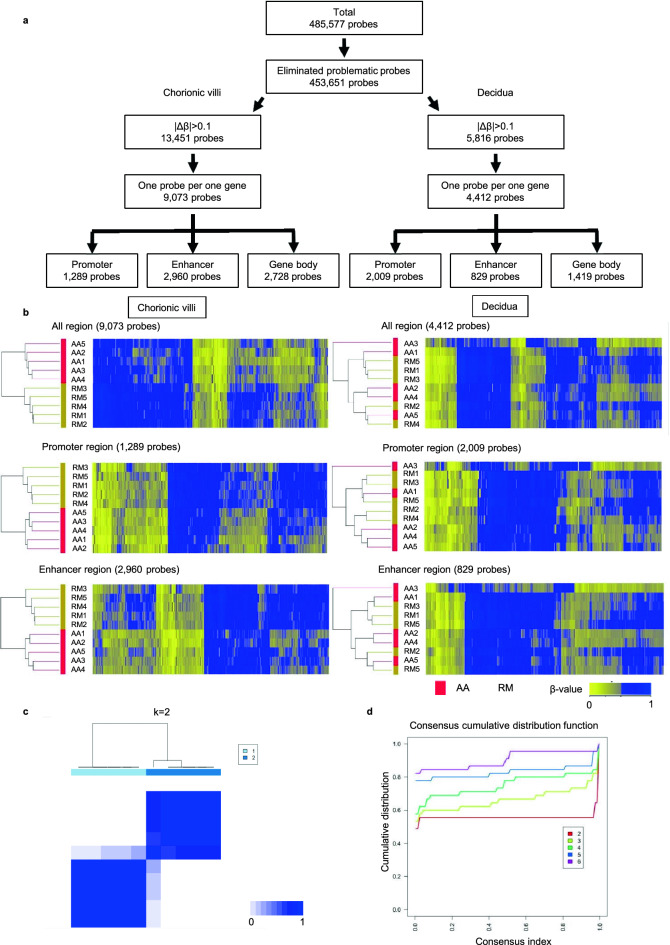
Genome-wide DNA methylation analysis. (**a**) Schema for the extraction of probes for unsupervised two-way hierarchical cluster analysis. Problematic probes indicate probes with missing values due to inadequate hybridization with an extremely low signal and with the SNPs at minor allele frequency > 0.1. |Δβ|, absolute difference in the average β-value between recurrent miscarriage and artificial abortion. SD, standard deviation. (**b**) Heat maps of unsupervised two-way hierarchical cluster analysis of 10 samples (recurrent miscarriage (RM); n = 5, artificial abortion (AA); n = 5) using differentially methylated probes in the chorionic villi and the decidua. The cluster analysis was performed for differentially methylated probes on all regions (9073 and 4412 probes), and separately for promoter regions (1289 and 2009 probes) and enhancer regions (2960 and 829 probes) in chorionic villi and decidua, respectively. Colors correspond to β-values as indicated (zero means a site is completely unmethylated while one means it is completely methylated). In the sample column, beige and red indicate recurrent miscarriage and artificial abortion, respectively. (**c**) Consensus clustering matrix of 9073 differentially methylated probes in chorionic villi for k = 2. Consensus index values range from 0 (higher dissimilar) to 1 (higher similar). (**d**) Cumulative distribution function plots from the consensus matrices for k = 2 to k = 6.

Of the original 485,577 Illumina Infinium HumanMethylation450 BeadChip probes, a total of 453,651 CpG probes that passed the quality control procedure was further analyzed (Fig. 1a, Materials and Methods). Of these, 13,451 and 5816 probes showed different β-values between RM and AA (DMPs) in the chorionic villi and decidua, respectively (Supplementary Tables S3, S4). Because there were multiple probes were designed within each gene, one representative probe showing the highest CpG score was selected^19^. This yielded a final count of 9073 DMPs and 4412 DMPs in chorionic villi and decidua, respectively, which were used for cluster analysis.

These DMPs were divided into three groups according to the location of their probes in each gene (i.e. promoter, enhancer, and gene body regions). Unsupervised two-way hierarchical cluster analysis using all DMPs showed clearly distinct DNA methylation signatures between RM and AA in chorionic villi but not in decidua (Fig. 1b). In addition, this clear difference only in chorionic villi was consistently observed when separately comparing DMPs corresponding to either promoter regions, enhancer regions, or gene body regions (Fig. 1b and Supplementary Fig. S2). Consistently, both K-means clustering analysis and consensus cumulative distribution function analysis revealed that DNA methylation profiles of all DMPs could be divided into two groups, depending on whether the chorionic villi samples were from RM patients or AA controls (Fig. 1c,d, and Supplementary Fig. S3). Notably, the ratio of male and female embryos used for genome-wide DNA methylation analysis was different between AA and RM samples (Supplementary Table S2). However, the clear differences in DNA methylation profiles between chorionic villi from RM and AA indicate that the aberrant epigenetic signature in chorionic villi but not in decidua is involved in developmental processes during pregnancy in RM patients.

### DNA methylation target genes in RM patients are enriched in specific pathways

Gene Ontology (GO) analysis was conducted to examine the biological pathways in which the genes differently methylated between chorionic villi of RM and AA in promoter and enhancer regions were involved. Among the 172 enriched GO terms, the most significant pathways in DMPs within regulatory regions (i.e. promoter and enhancer regions) were related to development-associated processes, cell membranous functions, and signal transduction-associated activities in the biological process (BP), cellular component (CC), and molecular function (MF) categories, respectively (Supplementary Table S5).

### DNA methylation analysis of frequently methylated genes in RM

GO analysis revealed that differentially methylated genes in regulatory regions are related to many developmental pathways. Active and poised enhancer elements, which are known to be regulated by DNA methylation, are particularly important for the transcriptional networks in cell type- and tissue-specific manners^20,21^. We analyzed DMPs in the enhancer regions to identify those genes that were consistently methylated in the chorionic villi of RM patients.

**Figure 2:**
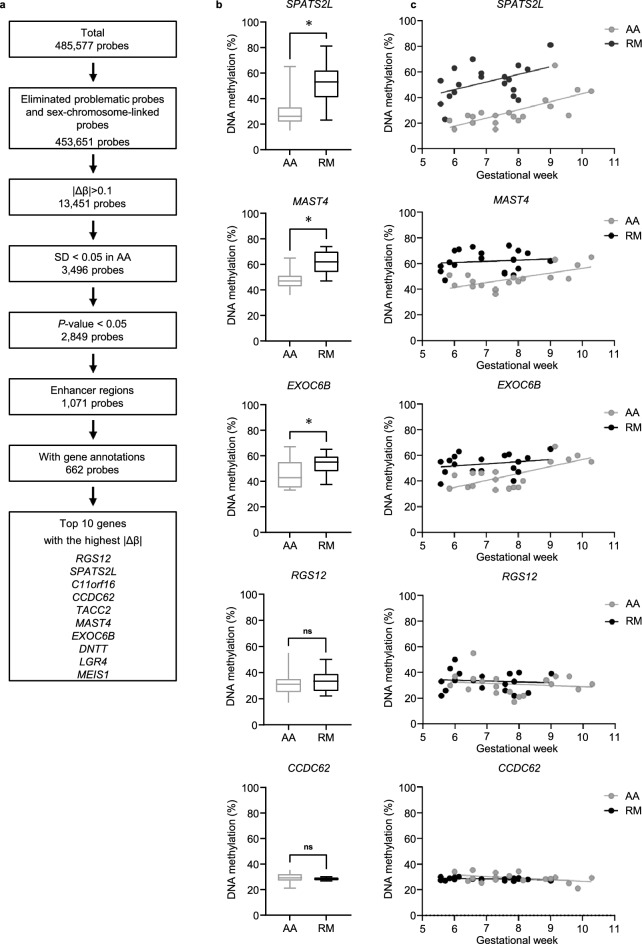
Genes differently methylated between recurrent miscarriage and artificial abortion. (**a**) Schema for the extraction of probes for targeted methylation analysis with pyrosequencing. Problematic probes indicate probes with missing values due to inadequate hybridization with an extremely low signal and with the SNPs at minor allele frequency > 0.1. |Δβ|, absolute difference in the average β-value between recurrent miscarriage (RM) and artificial abortion (AA). SD, standard deviation. (**b**) DNA methylation levels of *SPATS2L*, *MAST4*, *EXOC6B, RGS12,* and *CCDC62* (artificial abortion (AA); n = 19, recurrent miscarriage (RM); n = 19). The X-axis indicates sample group. The mean is indicated by a bold line inside the box the ends of which denote the upper and lower quartiles. Error bars represent the 5th and 95th percentile values. *, *P* value < 0.05. (**c**) Scatter plot of the DNA methylation level and gestational weeks for *SPATS2L*, *MAST4*, *EXOC6B, RGS12,* and *CCDC62*. The x-axis indicates gestational week. DNA methylation levels of each region are indicated on the y-axis.

The following criteria were used to identify candidate probes within enhancer regions: an absolute difference in the average β-value between RM and AA (|Δβ|) > 0.1, standard deviation (SD) within AA < 0.05 (i.e. DNA methylation status is consistent with the normal controls), *P* value < 0.05. This resulted in the identification of 552 candidate genes corresponding to 662 such probes (Fig. 2a).

The top 10 genes with the highest |Δβ| are listed. These represent those with the most significant differences in β-value between RM and AA (Table 2). For these genes, we designed 5 quantitative pyrosequencing DNA methylation assays, including the top two genes, namely, regulator of G protein signaling 12 (*RGS12*) and spermatogenesis associated serine rich 2 like (*SPATS2L*), corresponding to the differently methylated probe regions.

**Table 2:** Lists of candidate genes for targeted DNA methylation analysis (|Δβ|> 0.1).

Probe ID	Gene	Mean β value in RM patients	Mean β value in AA controls	|Δβ|	*P* value
cg03132824	*RGS12*	0.284	0.528	0.244	0.004
cg14106933	*SPATS2L*	0.266	0.509	0.243	< 0.001
cg14402562	*C11orf16*	0.659	0.420	0.239	0.002
cg05771701	*CCDC62*	0.364	0.144	0.220	0.017
cg23304023	*TACC2*	0.291	0.498	0.207	0.005
cg17462107	*MAST4*	0.351	0.556	0.205	< 0.001
cg09023965	*EXOC6B*	0.419	0.621	0.202	0.004
cg18278519	*DNTT*	0.326	0.527	0.201	< 0.001
cg24321971	*LGR4*	0.320	0.518	0.198	< 0.001
cg01863674	*MEIS1*	0.779	0.585	0.194	0.004

|Δβ|, absolute difference in the average β-value between recurrent miscarriage and artificial abortion. *RM* recurrent miscarriage; *AA* artificial abortion.

Of the five genes (*RGS12, SPATS2L,* coiled-coil domain containing 62 (*CCDC62*)*,* microtubule associated serine/threonine kinase family member 4 (*MAST4*)*,* exocyst complex component 6B (*EXOC6B*)), DNA methylation levels of *SPATS2L*, *MAST4*, and *EXOC6B* were significantly higher in chorionic villi from RM patients (n = 19) than AA controls (n = 19, *P* < 0.01) (Fig. 2b, Table 1). In contrast, DNA methylation levels of *RGS12* and *CCDC62* were not significantly different between RM patients and AA controls. We compared the DNA methylation levels of these genes in chorionic villi between male and female and found no significant differences between them (Supplementary Fig. S4).

Interestingly, there was a clear positive correlation between DNA methylation levels and gestational weeks in *SPATS2L* in both RM and AA groups (R^2^ = 0.217 and R^2^ = 0.485 in RM and AA, respectively) (Fig. 2c). Notably, mean DNA methylation levels of *SPATS2L* in RM at 6 weeks of gestation was about 40% (44 ± 11.2), almost the same as at 10 weeks in AA controls (38.7 ± 8.7) (*P* = 0.39). This suggests dysregulation of epigenetic processes during gestation in RM patients. In contrast, there was no correlation between the DNA methylation levels and the gestational week in *MAST4* or *EXOC6B* in either RM patients or AA controls.

### Reduced expression of SPATS2L as a common target of DNA methylation in RM patients

**Figure 3:**
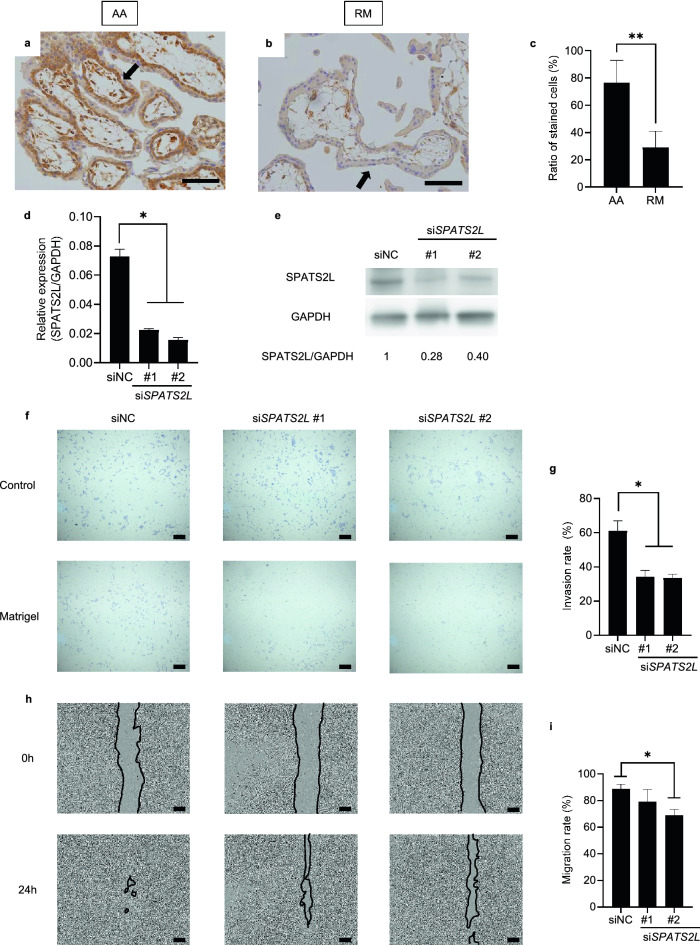
SPATS2L expression in chorionic villi samples. (**a**) and (**b**) Immunohistochemical analysis of SPATS2L in chorionic villi of artificial abortion (AA), and recurrent miscarriage (RM) samples. Scale bar indicates 100 μm. Arrows indicate representative cytotrophoblast cells. (**c**) Percentage of SPATS2L-positive cytotrophoblast cells (n = 14 AA, 12 RM). Error bars indicate the SD. **, *P* value < 0.01. (**d**) Expression of *SPATS2L* after treatment of HTR-8/SVneo cells with the si-RNA negative control (siNC) or si*SPATS2L* #1 and #2. Relative mRNA expression levels were normalized to GAPDH mRNA levels and are indicated on the y-axis. Error bars indicate the SD. *, *P* value < 0.05. (**e**) Protein expression by Western blotting. GAPDH protein was used as a loading control. Band densities were calculated and are indicated below the Western blots as relative values (SPATS2L/GAPDH). (**f**) Effect of si*SPATS2L* on the invasive ability of HTR-8/SVneo cells. Controls indicate wells with no matrigel basement membrane, while matrigel indicates wells with matrigel basement membranes. Scale bar indicates 200 μm. (**g**) Cell invasion rate of HTR-8/SVneo cells. Error bars indicate the SD. *, *P* value < 0.05. (**h**) Effect of si*SPATS2L* on migration of HTR-8/SVneo cells. The images are at 0 h (immediately after scratch) and 24 h. The black lines show the areas without cells. Scale bar indicates 200 μm. (**i**) The migration rate of HTR-8/SVneo cells. Error bars indicate the SD. *, *P* value < 0.05.

Because a significantly higher level of DNA methylation was detected in the enhancer region of *SPATS2L* in chorionic villi of RM patients than in AA controls, we quantified the level of SPATS2L protein therein. We found that this protein is expressed mainly in the cytoplasm of cytotrophoblast cells (Fig. 3a) but at a lower level in RM patients than AA controls (Fig. 3b). The fraction of cytotrophoblast cells expressing SPATS2L was significantly lower in chorionic villi tissues from RM patients than AA controls (29.2% and 76.4%, respectively; *P* = 0.012) (Fig. 3c). These data indicate that aberrantly increased hypermethylation of *SPATS2L* earlier in gestation leads to inappropriate suppression of SPATS2L protein expression in cytotrophoblast cells in RM patients.

### SPATS2L suppresses migration and invasion of extravillous trophoblast cells

Cytotrophoblast cells are highly proliferative epithelial cells with the ability to differentiate into extravillous trophoblast cells^22^. During embryogenesis, the migration of extravillous trophoblast cells into the decidua and myometrium plays a critical role in ensuring the normal embryonic development^23^. We knocked down *SPATS2L* gene expression by two independent siRNAs, both of which efficiently suppressed SPATS2L at the protein level, in a human extravillous trophoblast cell line, HTR8/SVneo (Fig. 3d,e). The depletion of SPATS2L significantly reduced the invasive ability of these cells (*P* < 0.05) (Fig. 3f,g). Furthermore, the scratch assay revealed significantly decreased cell migration of SPATS2L-depleted cells (*P* < 0.05) (Fig. 3h,i). Taken together, these data imply that altered SPATS2L expression may affect the cell migration and invasion during the early development of the placenta.

## Discussion

In this study, we determined genome-wide DNA methylation profiles in chorionic villi and decidual tissues from patients with karyotypically normal RM and compared them with gestational week-matched AA controls. RM decidua (maternal origin) and chorionic villi (embryo/fetal origin) exhibited DNA methylation profiles different from the controls. Previous studies demonstrated that altered DNA methylation in both chorionic villi and decidua may contribute to or be a consequence of poor placental function in miscarriage^24^. In addition, in the present study, we demonstrated that the DNA methylation signatures linked with disease status were more prevalent in chorionic villi^11–13^ than decidua^14^ in RM patients. Since the ratio of male and female embryos used for genome-wide DNA methylation analysis was not equal between AA and RM samples, it might be possible that the global DNA methylation status was affected by sex of the embryos. Inkster et al*.* identified 87 autosomal CpG regions that showed different methylation levels between males and females in the term placenta^25^. However, among the differentially methylated 13,451 probes between RM and AA in chorionic villi, only four probes (0.03% of 13,451 DMPs; cg00416882, cg07586008, cg00285394, cg00793719, Supplementary Table S3) showed significantly different DNA methylation level in chorionic villi between male and female (|Δβ|> 0.1, FDR < 0.05). Therefore, the potential confounding effects from the sex appears to be minimal in the current analysis, although we could not entirely rule out such posibility. Nevertherless, the dysregulation of DNA methylation staus in the early embryonic development is closely associated with the incidence of karyotypically normal RM.

DNA methylation profiling of enhancer regions identified the *SPATS2L* gene as significantly hypermethylated in chorionic villi of RM patients relative to AA controls. The increased DNA methylation was associated with decreased levels of SPATS2L protein in cytotrophoblast cells in RM patients. *SPATS2L*, located on chromosome 2q33, is also known as stress granule and nucleolar protein (*SGNP*), which localizes within stress granules under oxidative stress conditions in certain types of cells, suggesting that it is involved in RNA metabolism^26^. *SPATS2L* was also reported as a bronchodilator response gene that may be an important regulator of β2 adrenergic receptor down-regulation^27^. However, detailed biological function data on this protein during development and gestational processes are largely absent. Here, we found that DNA methylation in the enhancer regions of *SPATS2L* resulted in decreased protein expression in chorionic villi. The human extravillous trophoblast cell line HTR8/SVneo is widely used as a model for first trimester extravillous trophoblasts invasion and migration^28–30^. Our functional study using this cell line revealed that SPATS2L is involved in its migration and invasion. Cytotrophoblasts are the primary placental cells which transform into extravillous trophoblast cells that migrate into the decidua and play a key role in placental development^31^. Previous studies showed that shallow or insufficient extravillous trophoblast migration is implicated in the etiology of pregnancy complications, including RM^31–33^. The present data indicate that an appropriate level of SPATS2L expression in cytotrophoblast and extravillous trophoblast cells may be required for development and establishment of the placenta at an early stage of pregnancy.

The reason why DNA methylation levels in the enhancer regions of SPATS2L were significantly higher in chorionic villi of RM patients than AA controls is uncertain. Enhancers are situated at variable distances from promoters and are key to controlling gene expression in development and cell function via epigenomic mechanisms including DNA methylation^20,34^. After fertilization, the embryonic DNA is demethylated in most regions, after which the cells in the inner cell mass undergo de novo methylation, while the trophectoderm cells remain hypomethylated^15^. The global DNA methylation levels of a set of genes increases along with gestational age also in trophectoderm cells^35,36^, suggesting that time-dependent dynamic epigenetic programs during early development are involved in placental development. Indeed, levels of DNA methylation of *SPATS2L* increased with increasing gestation week in both RM patients and AA controls, although baseline levels were significantly higher in the former, indicating that silencing of SPATS2L at an appropriate time might be required for a normal pregnancy.

Hanna et al. have proved that the DNA methylation differences exist in placental villi between RM and sporadic miscarriage instead of AA as controls^11^. In another study showed that CREB5 expression was dysregulated by aberrant DNA methylation in decidua from RM patients^14^. We found DNA methylation changes in the CREB5 genes in villi between RM and AA (Supplementrty Table S3), although the differences is substantilally smaller in comparison with SPATS2L. It remains to be determined whether altered DNA methylation is a causal mechanism for RM or a consequence of embryo demise. However, our genome-wide DNA methylation analysis that simultaneously examined the chorionic villi and decidual tissues derived from the same RM patients revealed clearly distinct disease-associated DNA methylation profiles in chorionic villi, but not in maternal decidua. This indicates the altered DNA methylation program in a particular tissues is involved in the maintenance of a successful pregnancy. Furthermore, dysregulation of an affected gene, *SPATS2L*, downregulated the function of extravillous trophoblast cells. Such epigenetic alterations may be induced in response to environmental factors on the background of certain genetic polymorphisms. Further studies with a larger, well-characterized population will allow us to better understand the epigenome-associated mechanism responsible for RM.

## Supplementary Information

Supplementary Information 1.

Supplementary Information 2.

Supplementary Information 3.

Supplementary Information 4.

Supplementary Information 5.

## Data Availability

The methylation microarray data is available through Gene Expression Omnibus (accession number GSE198700; https://www.ncbi.nlm.nih.gov/geo/query/acc.cgi?acc=GSE198700).
