# Supplementary material for: Characteristic DNA methylation profiles of chorionic villi in recurrent miscarriage
Source: Sci Rep. 2022 Jul 27;12:11673. doi: 10.1038/s41598-022-15656-y (PMC9329430; doi:10.1038/s41598-022-15656-y)
Supplement: Supplementary file 1 — Supplementary Information 1. [file 41598_2022_15656_MOESM1_ESM.pdf]

Supplementary Figure S1.

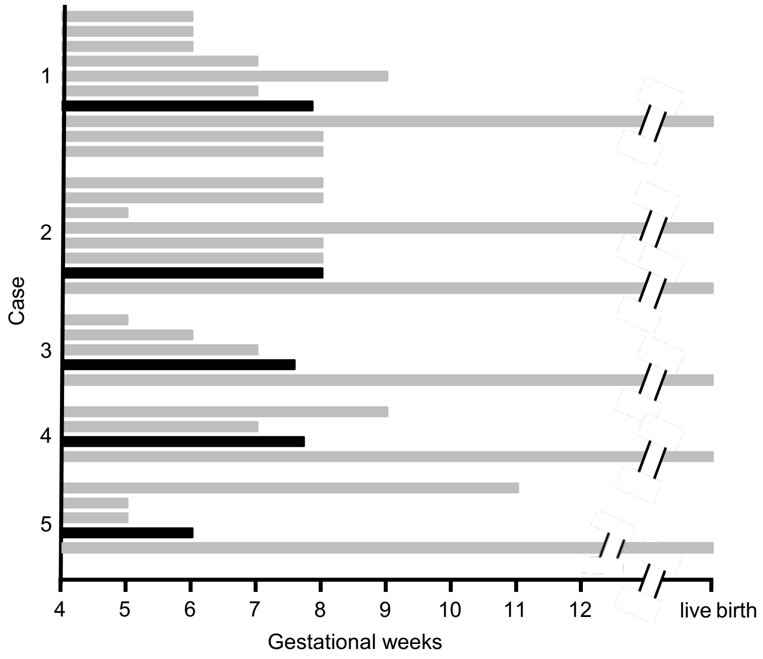

Supplementary Figure S1. Background of patients with recurrent miscarriage.

Clinical background of miscarriage in five patients with recurrent miscarriage. Each bar indicates each gestation from top to bottom. Samples that were analyzed for the genome-wide DNA methylation status are shown as black bars. X-axis indicates gestational weeks. Y-axis indicates each gestation in each patient.

Supplementary Figure S2.

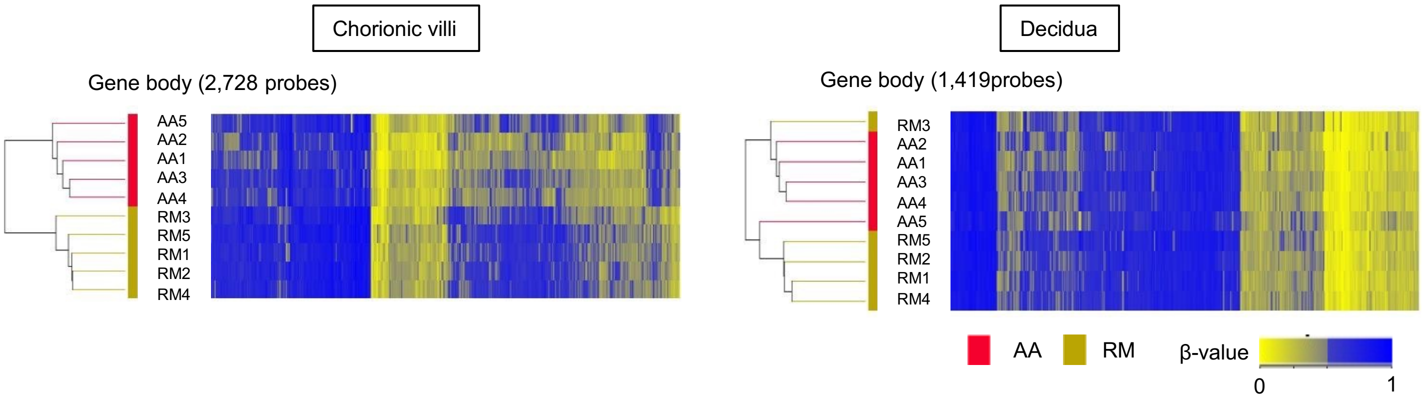

Supplementary Figure S2. **Heat maps of unsupervised two-way hierarchical cluster analysis.**

Heat maps of unsupervised two-way hierarchical cluster analysis of 10 samples (recurrent miscarriage (RM); n=5, artificial abortion (AA); n=5) using differentially methylated probes in the chorionic villi and the decidua. The cluster analysis was performed for differentially methylated probes on gene body (2,728 and 1,419 probes in chorionic villi and decidua, respectively). Colors correspond to β-values as indicated (zero means a site is completely unmethylated while one means it is completely methylated). In the sample column, beige and red indicate recurrent miscarriage and artificial abortion, respectively.

Supplementary Figure S3.

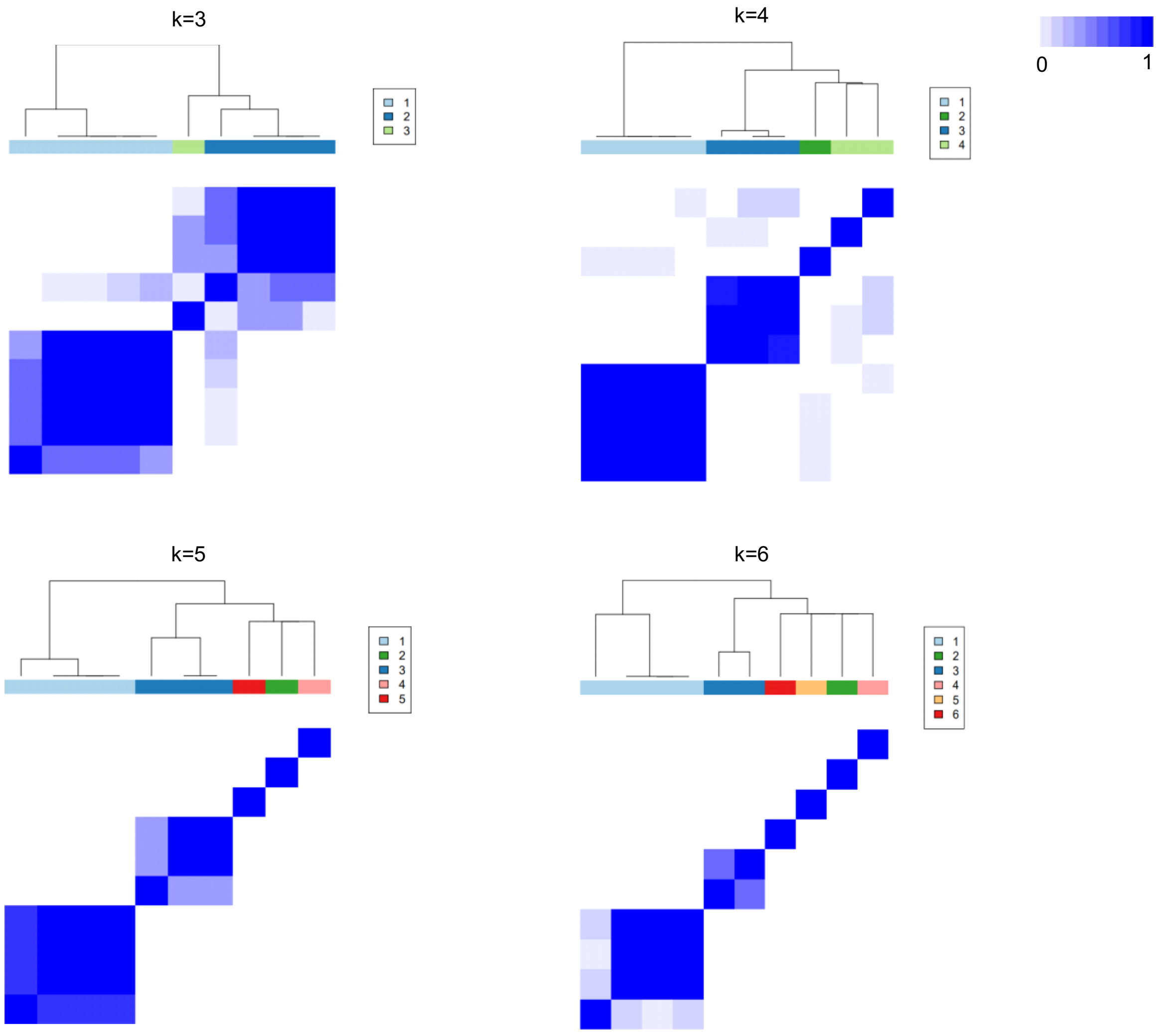

Supplementary Figure S3. Consensus clustering matrix of 9,073 differentially methylated probes in chorionic villi for k = 3, 4, 5 and 6.

Consensus index values range from 0 (higher dissimilar) to 1 (higher similar).

Supplementary Figure S4.

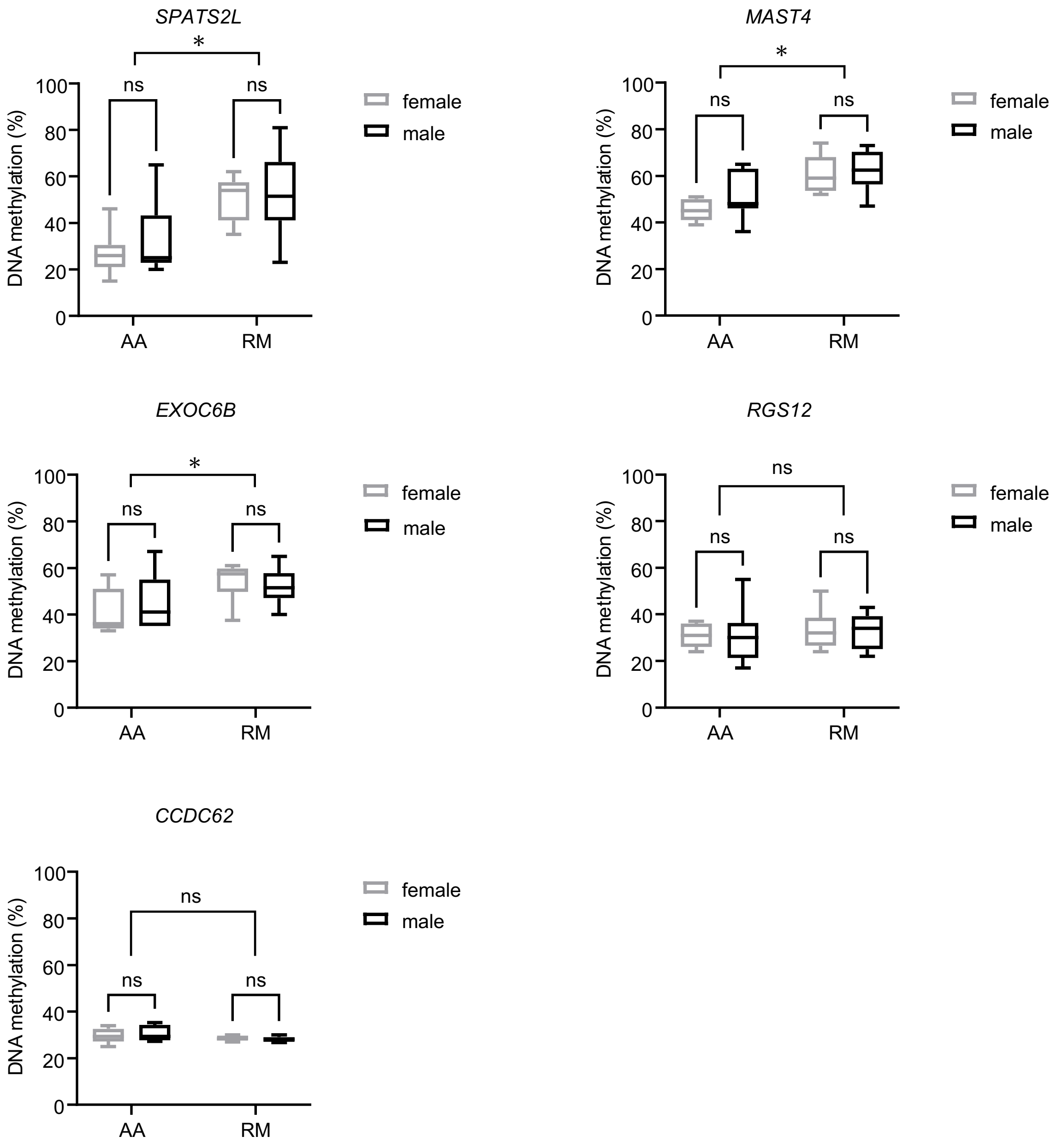

Supplementary Figure S4. DNA methylation levels of SPATS2L, MAST4, EXOC6B, RGS12, and CCDC62 according to sex.

DNA methylation levels of SPATS2L, MAST4, EXOC6B, RGS12, and CCDC62. Samples (artificial abortion (AA); n=17, recurrent miscarriage (RM); n=19) were divided into four groups according to sex (AA female; n=9, AA male; n=8, RM female; n=9, RM male; n=10). Tow AA samples used in Figure 2 were excluded because of unknown sex. The X-axis indicates sample group. The mean is indicated by a bold line inside the box the ends of which denote the upper and lower quartiles. Error bars represent the 5th and 95th percentile values. P-value was calculated by using two-way ANOVA. P-value < 0.05 was considered statistically significant. *, *P*-value < 0.05. ns, not statistically significant.
