# Supplementary material for: Characteristic DNA methylation profiles of chorionic villi in recurrent miscarriage
Source: Sci Rep. 2022 Jul 27;12:11673. doi: 10.1038/s41598-022-15656-y (PMC9329430; doi:10.1038/s41598-022-15656-y)
Supplement: Supplementary file 2 — Supplementary Information 2. [file 41598_2022_15656_MOESM2_ESM.pptx]

## Slide 1
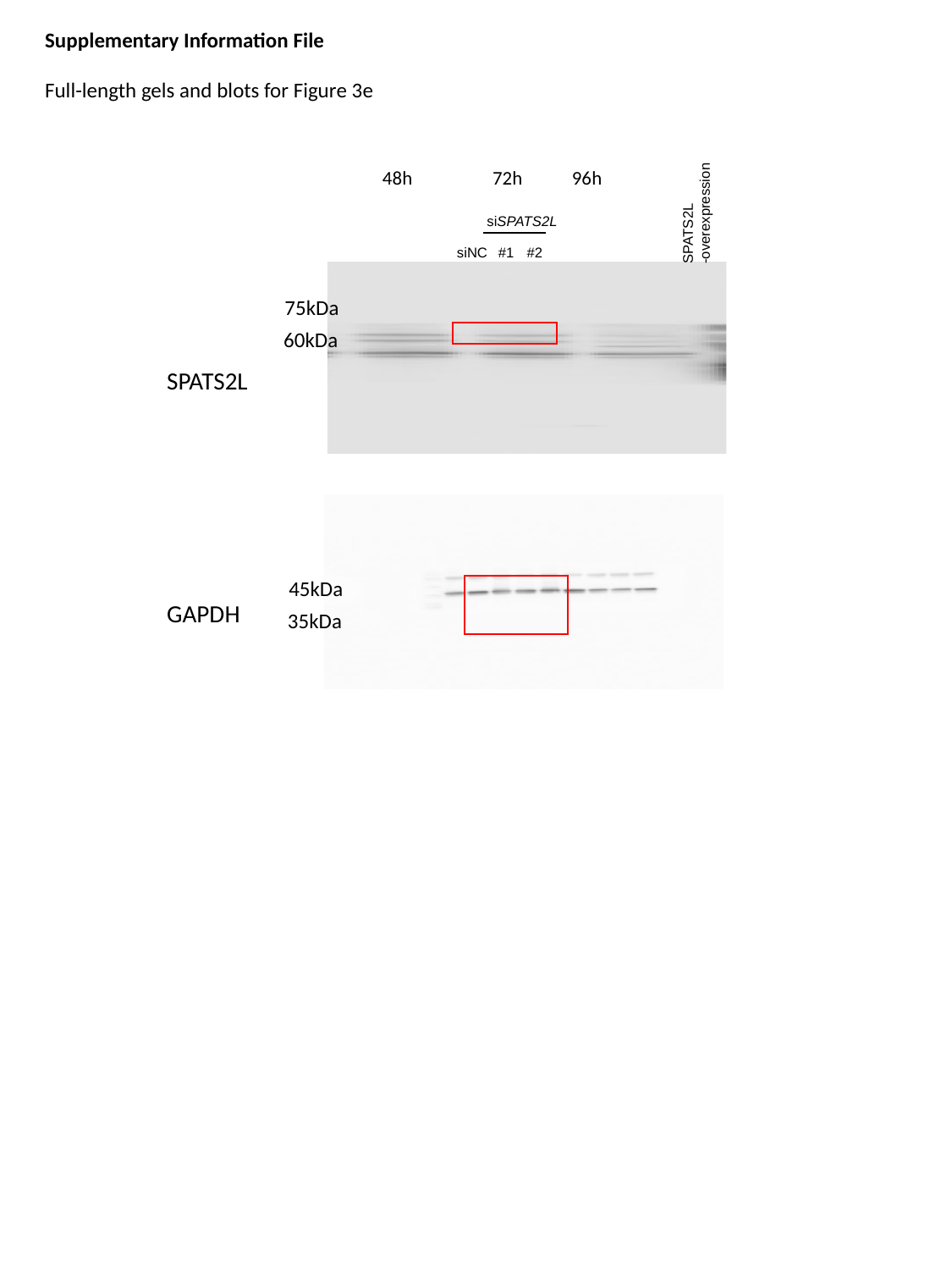

Supplementary Information File
Full-length gels and blots for Figure 3e
48h
72h
96h
SPATS2L
-overexpression
siSPATS2L
siNC
#1
#2
75kDa
60kDa
SPATS2L
45kDa
GAPDH
35kDa
