# Supplementary material for: Characteristic DNA methylation profiles of chorionic villi in recurrent miscarriage
Source: Sci Rep. 2022 Jul 27;12:11673. doi: 10.1038/s41598-022-15656-y (PMC9329430; doi:10.1038/s41598-022-15656-y)
Supplement: Supplementary file 3 — Supplementary Information 3. [file 41598_2022_15656_MOESM3_ESM.pdf]

**Supplementary Table S1.** Primer sequences

| Gene | Type | Sequence (5' to 3') |
|---|---|---|
| Pyrosequencing | | |
| *SPATS2L* | Forward | GGGACACCGCTGATCGTTTATAGTGGGGTTTGGTGGATGTAA |
| | Reverse | CCACCACTCTTAACCATAATTCTTTTCT |
| | Sequencing | ACTACTCTAAAAATCCACA |
| *MAST4* | Forward | GTTTGGATGGTTGGTGTTAGAT |
| | Reverse | GGGACACCGCTGATCGTTTACTTAAACTTTCCACTTTTACATT |
| | Sequencing | GTTGGATGGTTGGTGTTAGAT |
| *EXOC6B* | Forward | TGTGTGGTTTTGGTTATTAAAAAGTGA |
| | Reverse | GGGACACCGCTGATCGTTTAACTAAACAACATTACCCAAAATACAATA |
| | Sequencing | AAAAAGTGATTTAGGTTTGTA |
| *RGS12* | Forward | GTTTGTTGGGTAGGGTTAGTATG |
| | Reverse | GGGACACCGCTGATCGTTTACCCCCTACCCCAAAACAA |
| | Sequencing | GTTGTTTGGAAGGTGAAG |
| *CCDC62* | Forward | CAGTGTTAGGTTTTGTTTTGGGAGTTAGTT |
| | Reverse | GGGACACCGCTGATCGTTTAAATTACTCACTTCCCTATCTT |
| | Sequencing | GGGAGTTAGTTATATAGGAG |
| siRNA | | |
| si*SPATS2L* | Sense | GAAUGGAUCUUCUAACCAAtt |
| #1 | Antisense | UUGGUUAGAAGAUCCAUUCtg |
| si*SPATS2L* | Sense | CACAGAUUCUGCUAACGAAtt |
| #2 | Antisense | UUCGUUAGCAGAAUCUGUGga |
| qRT-PCR | | |
| *SPATS2L* | Forward | GTGGATGGCAGTGCAATTCA |
| | Reverse | AAGCAGCATCAAGGCAACAA |
| *GAPDH* | Forward | GGCACCGTCAAGGCTGAGAA |
| | Reverse | CCAGCATCGCCCCACTTGAT |

qRT-PCR, quantitative reverse transcription-PCR

Universal primer, Biotin-GGGACACCGCTGATCGTTTA

**Supplementary Table S2.** Detailed information on patients with recurrent miscarriage and control women with artificial abortion for genome-wide DNA methylation analysis

| **Group** | **Patient number** | **Age** | **Number of miscarriages** | **Number of previous live births** | **Gestational weeks** | **Karyotype of chorionic villi** |
|---|---|---|---|---|---|---|
| Recurrent miscarriage | 1 | 33 | 7 | 0 | 7w6d | 46, XY |
| | 2 | 38 | 6 | 1 | 8w0d | 46, XY |
| | 3 | 38 | 4 | 0 | 7w0d | 46, XY |
| | 4 | 30 | 3 | 0 | 7w5d | 46, XX |
| | 5 | 35 | 4 | 0 | 6w0d | 46, XY |
| Artificial abortion | 1 | 27 | 0 | 0 | 6w1d | 46, XX |
| | 2 | 34 | 0 | 5 | 5w6d | 46, XX |
| | 3 | 39 | 0 | 3 | 8w0d | 46, XY |
| | 4 | 21 | 0 | 0 | 7w5d | 46, XX |
| | 5 | 42 | 0 | 2 | 6w4d | 46, XX |

**Supplementary Table S3. All DMPs of chorionic villi**

See txt. file

**Supplementary Table S4. All DMPs of decidua**

See txt. file

**Supplementary Table S5.** The 10 gene ontology (GO) terms from the lowest *P*-value in the biological process (BP), cellular components (CC) and molecular function (MF) categories analyzed with the probes of chorionic villi in promoter regions and enhancer regions.

Promoter

| Class | GO Term | *P*-value |
|---|---|---|
| BP | system process | 1.75E-07 |
| | multicellular organismal process | 5.58E-06 |
| | neurological system process | 4.37E-05 |
| | response to lipopolysaccharide | 6.09E-05 |
| | response to molecule of bacterial origin | 6.67E-05 |
| | transmembrane transport | 7.88E-05 |
| | G-protein coupled receptor signaling pathway | 1.22E-04 |
| | response to lipid | 1.42E-04 |
| | sensory perception | 2.16E-04 |
| | single-organism process | 3.21E-04 |
| CC | cell periphery | 4.95E-07 |
| | plasma membrane | 3.19E-06 |
| | integral component of plasma membrane | 6.46E-05 |
| | plasma membrane part | 6.50E-05 |
| | intrinsic component of plasma membrane | 1.05E-04 |
| | intrinsic component of membrane | 4.42E-04 |
| | integral component of membrane | 4.51E-04 |
| | membrane part | 7.54E-04 |
| | actin cytoskeleton | 0.0013 |
| | secretory granule membrane | 0.0017 |
| MF | signal transducer activity | 2.49E-04 |
| | signaling receptor activity | 0.001 |
| | molecular transducer activity | 0.0011 |
| | receptor activity | 0.0011 |
| | olfactory receptor activity | 0.0011 |
| | transmembrane signaling receptor activity | 0.0011 |
| | transmembrane receptor activity | 0.0013 |
| | transmembrane transporter activity | 0.0015 |
| | G-protein coupled receptor activity | 0.0022 |
| | 3',5'-cyclic-nucleotide phosphodiesterase activity | 0.0027 |

Enhancer

| Class | GO Term | *P*-value |
|---|---|---|
| BP | system development | 1.51E-17 |
| | anatomical structure development | 2.06E-16 |
| | single-multicellular organism process | 2.30E-16 |
| | developmental process | 7.24E-16 |
| | cellular developmental process | 1.21E-15 |
| | single-organism developmental process | 3.59E-15 |
| | cell differentiation | 4.51E-15 |
| | signaling | 5.36E-15 |
| | multicellular organism development | 9.91E-15 |
| | cell communication | 1.50E-14 |
| CC | cell junction | 6.14E-14 |
| | plasma membrane part | 1.30E-09 |
| | cell projection | 2.07E-09 |
| | synapse | 6.52E-09 |
| | cytoplasm | 1.31E-08 |
| | cell periphery | 1.86E-08 |
| | plasma membrane | 4.10E-08 |
| | neuron part | 4.26E-08 |
| | plasma membrane region | 1.85E-07 |
| | adherens junction | 3.39E-07 |
| MF | phosphotransferase activity, alcohol group as acceptor | 3.39E-08 |
| | carbohydrate derivative binding | 1.30E-07 |
| | kinase activity | 1.37E-07 |
| | adenyl ribonucleotide binding | 6.58E-07 |
| | adenyl nucleotide binding | 9.62E-07 |
| | ATP binding | 2.19E-06 |
| | purine ribonucleotide binding | 2.39E-06 |
| | ribonucleotide binding | 2.55E-06 |
| | protein kinase activity | 3.29E-06 |
| | purine nucleotide binding | 3.45E-06 |
